# Finite element analysis and bearing capacity calculation of cross-shaped CFST columns under compressive load

**DOI:** 10.1016/j.heliyon.2024.e28715

**Published:** 2024-03-26

**Authors:** Mingjian Yang, Desun Yu, Qirong Qiu, Yong Yu

**Affiliations:** a Jiujiang Vocational and Technical College, Jiujiang, 332007, China; b Shanghai Construction Engineering Fifth Construction Group Co., Ltd. Shanghai, 200063, China; c School of Civil Engineering and Engineering Management, Guangzhou Maritime University, Guangdong, 510725, China

**Keywords:** Cross-shaped CFST column, Eccentric compression, Axial compression, Finite element, Bearing capacity calculation

## Abstract

The study investigated the load capacity of cross-shaped concrete-filled steel tubular (CFST) columns under axial and eccentric compression using finite element software ABAQUS. It analyzed six specimens with measured data and an additional 26 specimens with varied parameters, including eccentricity, slenderness ratio, section steel ratio and material properties such as concrete strength and steel yield strength.The objective was to understand how these parameters affect the load capacity of cross-shaped CFST columns. The research findings suggest that as eccentricity and slenderness ratio increase, the ultimate capacity decreases. Conversely, it increases with higher steel content, concrete strength and steel yield strength. Moreover, the bearing capacity deteriorates more rapidly with reduced eccentricity and concrete strength, while it demonstrates a nearly linear increase with greater steel content. Additionally, the study found that enhancing the resilience of the channel steel significantly boosts the load-bearing capacity of the column. Based on these findings, practical design equations were developed to determine the maximum bearing capacity of cross-shaped CFST columns under axial and eccentric compression. These equations are grounded in confined concrete theory and demonstrate robust applicability for practical design purposes.

## 1 Introduction

The uniquely shaped column structures, such as L-shaped, T-shaped, cross-shaped and other specialized cross-sections, offer significant advantages over traditional rectangular columns. They optimize useable area and aesthetics by avoiding protrusions into the room, making them widely favored in engineering projects [[1], [2], [3], [4], [5]]. However, with increasing building demands, these special-shaped columns must support heavier loads. Unfortunately, conventional reinforced concrete (RC) special-shaped columns face limitations in both load capacity and seismic performance due to their slender legs, thereby restricting their use in super-high buildings and regions prone to high seismic activity.

Concrete-filled steel tube (CFST) structures offer a multitude of structural advantages, including robust strength, fire resistance, flexibility and efficient energy absorption. Moreover, the elimination of formwork support during concrete pouring reduces construction time and expenses. These advantages have led to the widespread adoption of steel-concrete structures in civil engineering [[6], [7], [8], [9]]. Scholars have also introduced special-shaped columns made of steel tubes filled with concrete, aiming to further enhance the application of CFST columns in building structures. Extensive tests, theoretical studies and finite element analyses worldwide have explored the mechanical properties of L [10,11], T [[12], [13], [14]] and cross-shaped [[15], [16], [17], [18], [19]] CFST columns. These investigations consistently demonstrate the excellent load capacity and seismic performance of CFST special-shaped columns, with the cross section being particularly prevalent. While existing literature extensively covers construction methods for CFST cross-section columns, such as steel tubes combined with battens or other connections, research on integrating steel tubes with channel steel remains limited. In Ref. [20], eccentric compression tests were conducted on six cross-shaped CFST columns, revealing that this composite configuration effectively constrained the core concrete and exhibited promising mechanical properties. However, due to the limited number of test specimens, gaining a comprehensive understanding of its internal behavior proved challenging. Consequently, further comprehensive and systematic research is warranted.

To comprehensively assess the load-bearing capacity of cross-shaped CFST structures employing a combination of steel tube and channel steel, this study builds upon the findings of reference [20] and incorporates successful test and finite element simulation outcomes. Leveraging our research expertise, we conduct an in-depth analysis of 22 specimens, consisting of 6 tested and measured samples along with 16 numerically simulated ones. Our analysis investigates the influence of critical factors such as eccentricity, slenderness ratio, section steel ratio and concrete strength grade on the load-bearing capacity of cross-shaped CFST structures. Additionally, we propose calculation methodologies for both axial and eccentric compression scenarios.

## 2 Experimental program

**Fig. 1:**
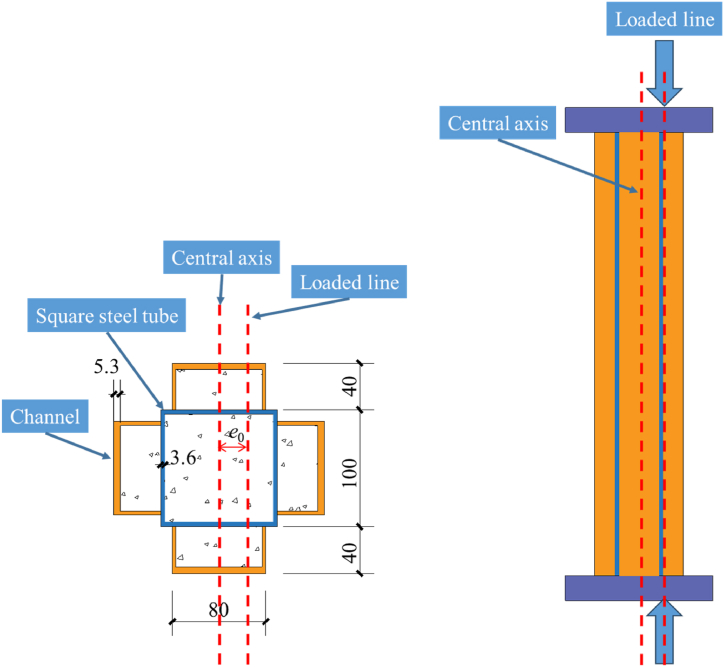
Cross section diagram of composite cross shaped column.

In reference [**20**], six cross-shaped CFST eccentrically compressed columns were constructed with eccentricity (*e*_0_) and slenderness ratio (*λ*) as variable parameters. The column design is depicted in Fig. 1, and the detailed design variables of the test specimen are displayed in Table 1. The square steel tube utilized in the experiment measures 100 mm in side length, with a thickness of 3.6 mm and a yield strength of 338 MPa. The channel steel dimensions are represented as *H* × *B* × *t*_w_ × *h*_w_, where *H* = 80 mm, *B* = 40 mm, *t*_w_ = 5.3 mm and *h*_w_ = 3.1 mm, possessing a yield strength of 458 MPa. Notably, in the experiment, the channel profile was not welded to the box to prevent premature buckling [21]. The compressive strength of the concrete cube used is *f*_c_ = 49.1 MPa.

**Table 1:** Experimental parameters and test results.

Column no.	*e*_0_ (mm)	*L* (mm)	*λ*	*P*_t_ (kN)	*P*_c_ (kN)	*P*_t_/*P*_c_
spa-25	25	500	9.44	1871	1737	1.08
spa-50	50	500	9.44	1450	1420	1.02
spb-25	25	700	13.22	1842	1869	0.99
spb-50	50	700	13.22	1254	1290	0.97
spc-25	25	900	17.00	1607	1566	1.03
spc-50	50	900	17.00	1190	1099	1.08

Note: *e*_0_ is the eccentricity. *L* is the column length. *λ* represents the shear span ratio. *P*_t_ is the ultimate capacity. *P*_c_ is the peak load calculated by finite element method.

## 3 Finite element computational model

### 3.1 Material constitutive models

#### 3.1.1 Steel

**Fig. 2:**
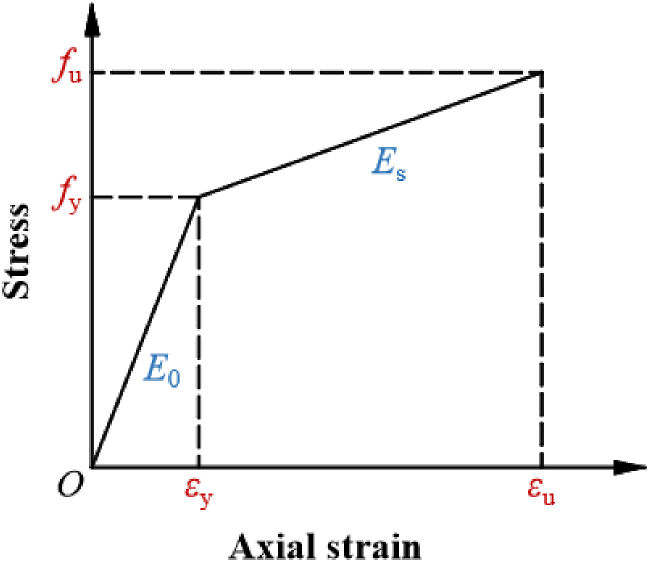
Elastic-plastic model.

The structural steel's constitutive relationship in ABAQUS/CAE employs the strain hardening bilinear model, neglecting the substantial deformation of materials. It is assumed that the actual stress and strain of the material directly correspond to the nominal ones, respectively. The full-range stress-strain relation of the steel can be visualized in Eq. (1) and Fig. 2 [22,23].

(1) $$\sigma =\left\{ \begin{array}{c} E_{0}\varepsilon \;\left(0\leq \varepsilon \leq \varepsilon_{y}\right)\\ f_{y}+E_{s}\left(\varepsilon -\varepsilon_{y}\right)\;\left(\varepsilon_{y}＜\varepsilon \right) \end{array}\right.$$

In this context, *f*_y_ and *ε*_y_, respectively, denote the yield strength and yield strain. Similarly, *f*_u_ and *ε*_u_ represent the maximum strength and peak strain. *E*_0_ signifies the modulus of elasticity, whereas *E*_s_ indicates the hardening stiffness, with *E*_s_ being equal to 0.01 times *E*_0_.

#### 3.1.2 Concrete

Two methodologies have been delineated in the extant literature to emulate constrained concrete columns. One approach entails the direct application of the constitutive relation governing constrained concrete, which encompasses augmentations in concrete material characteristics. Conversely, an alternative strategy involves employing a uniaxial constitutive model for concrete, with automated software mechanisms for constraint consideration. Given the specific focus on square steel tube buckling and the interface between concrete and square steel tubes in this discourse, a uniaxial stress model has been selected. The ABAQUS software has utilized the concrete damage plasticity model (CDP) for analysis. The constitutive equation for concrete as delineated in GB50010-2010 ″Code for Design of Concrete Structures" [24] has been adopted herein, with the computational formulas presented in Eqs. (2), (3)). Additionally, the friction angle and viscosity coefficient of concrete were, respectively, established at 30° and 5 × 10^−4^.

(2) $$y=\left\{ \begin{array}{c} \alpha_{a}x+\left(3-2\alpha_{a}\right)x^{2}+\left(\alpha_{a}-2\right)x^{3}\;0\leq x\leq 1\\ \frac{x}{\alpha_{d}{\left(x-1\right)}^{2}+x}\;x>1 \end{array}\right.$$

(3) $$y=\frac{\sigma }{f_{c}},x=\frac{\varepsilon }{\varepsilon_{c}}$$

Here, *f*_c_ and *ε*_c_, respectively, represent the concrete's maximum compression stress and strain. *α*_a_ and *α*_d_ signify the parameters of the ascending and descending segments, respectively.

### 3.2 Finite element mesh

The columns are primarily composed of concrete, channel steel together with square steel tube [[25], [26], [27], [28]]. Dimensional consistency between the numerical simulation and experimental models is ensured. Moreover, the simulation presupposes zero slippage between the structural steel, reinforcement and concrete, essentially integrating both the steel and reinforcement within the concrete matrix. Please refer to Table 2 for specific information on the selected element types for different components.

**Table 2:** Selection of element type.

Materials	Concrete	Steel tube	Channel steel
Strength grade	C40	Q335	Q335
Selected element	C3D8R	S4R	S4R

### 3.3 Boundary conditions

**Fig. 3:**
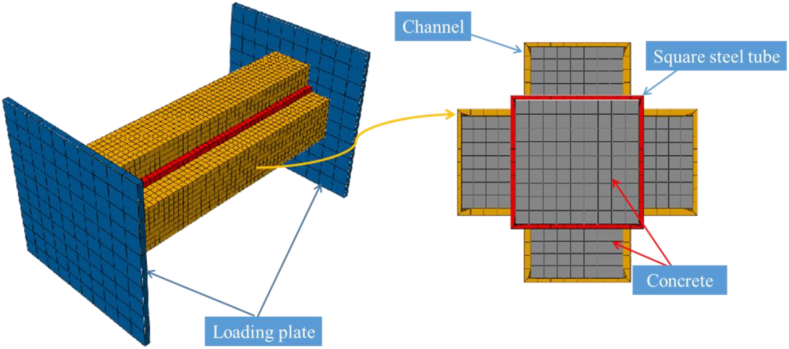
Computational model.

During the construction of the seismic test's numerical simulation model, it's possible to eliminate the loading device. Alternatively, a coupling reference point can be established at the apex of the column to concomitantly constrain both the torsion angle and horizontal displacement. The final numerical simulation model is depicted in Fig. 3. Notably, for clarity purposes, the thickness of the shell element was emphasized in the figure. Therefore, although visually appearing as solid, it should be noted that it is, in reality, a shell element.

### 3.4 Model verification

**Fig. 4:**
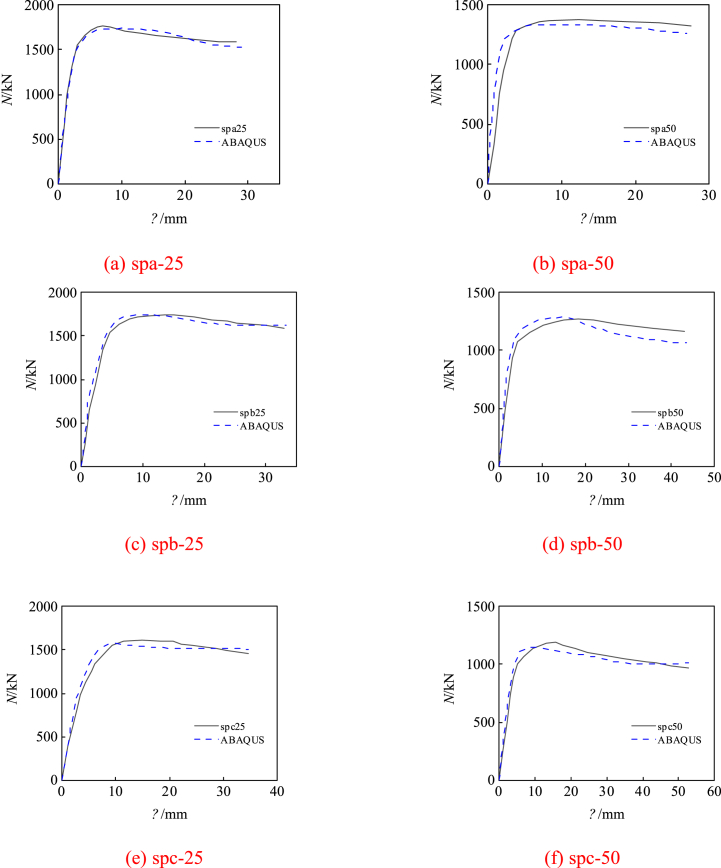
Comparing experimental and simulated load-displacement curves.

**Fig. 5:**
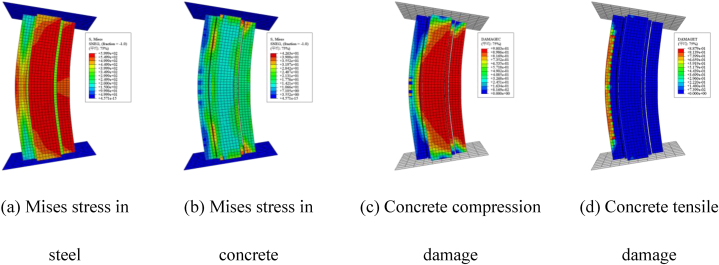
Stress and damage cloud map of the specimen.

**Fig. 6:**
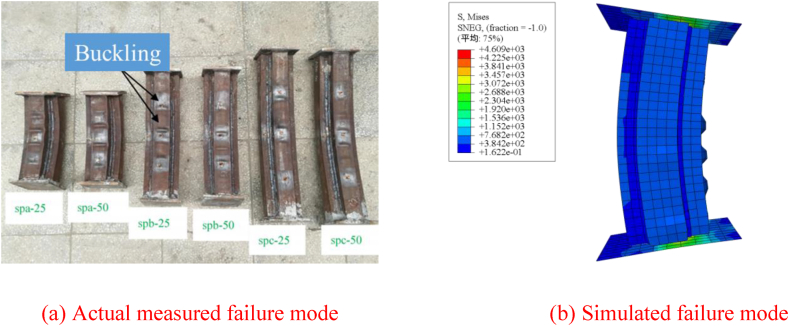
Local buckling of steel tubes.

The comparison of load-deflection curves for the six specimens, as calculated using the modeling techniques and material properties outlined in Ref. [16], is presented in Fig. 4a–f. As observed in the figures, the trends of the load-deflection curves obtained from finite element simulation closely resemble the experimental results. Fig. 5a–d displays stress contour plots for selected finite element models, revealing significant stress distribution on both the compression and tension sides of the channel steel, indicating good plasticity. Furthermore, evident compression/tension damage in the concrete highlights a substantial increase in concrete bearing capacity during eccentric compression. Fig. 6a and b compares failure modes, with simulated results showing local bulging of the steel tube, consistent with experimental observations. It's important to note that the buckling morphology of some specimens doesn't fully match the simulation results due to idealization of the numerical model and inadequate consideration of loading errors during experimentation. Furthermore, the interaction dynamics between steel pipes and concrete solely factor in contact, posing challenges in accommodating adhesion and detachment phenomena between the steel tube and concrete. Nonetheless, the pronounced resemblances in curves and failure modes underscore the necessity for comprehensive scrutiny and merit further investigation.

Table 1 provides a comprehensive comparison of the ultimate bearing capacity. It elucidates that the average ratio of ultimate bearing capacity between the finite element simulations and experimental findings is 1.027. The variance is recorded at 0.045, accompanied by a coefficient of variation of 0.044. These findings affirm the reliability of the finite element model devised in this study for predicting the mechanical response of the cross-shaped CFST column under eccentric compression. Following the simulation analysis findings, the spc-50 specimen, exhibiting a significant alignment between the failure mode and the curve, was selected as the reference specimen for extended parameter analysis. Table 3 provides comprehensive information on the parameters and calculation outcomes of all extended specimens, encompassing variables such as eccentricity *e*_0_, slenderness ratio *L*, steel tube thickness *t*, concrete strength grade, steel tube yield strength *f*_yt_ and channel steel yield strength *f*_yc_.

**Table 3:** Comparison of finite element results and experimental findings.

Specimen No.	*e*_0_ (mm)	*L* (mm)	*λ*	*t* (mm)	Steel content *ρ*	Concrete strength grade	*f*_yt_ (MPa)	*f*_yc_ (MPa)	*P* (kN)
spc-50	50	900	17.00	3.6	17.3%	C45	338	458	1190
CFST-1	70	900	17.00	3.6	17.3%	C45	338	458	1155
CFST-2	80	900	17.00	3.6	17.3%	C45	338	458	1077
CFST-3	90	900	17.00	3.6	17.3%	C45	338	458	992
CFST-4	100	900	17.00	3.6	17.3%	C45	338	458	921
CFST-5	0	900	17.00	3.6	17.3%	C45	338	458	2473
CFST-6	0	1200	22.66	3.6	17.3%	C45	338	458	2410
CFST-7	0	1500	28.33	3.6	17.3%	C45	338	458	2158
CFST-8	0	1800	34.00	3.6	17.3%	C45	338	458	2026
CFST-9	0	3500	66.00	3.6	17.3%	C45	338	458	1953
CFST-10	0	4500	85.00	3.6	17.3%	C45	338	458	1706
CFST-11	0	5500	103.77	3.6	17.3%	C45	338	458	1384
CFST-12	0	6500	122.64	3.6	17.3%	C45	338	458	1088
CFST-13	0	900	17.00	2	14.6%	C45	338	458	2224
CFST-14	0	900	17.00	3	16.3%	C45	338	458	2379
CFST-15	0	900	17.00	4	17.9%	C45	338	458	2534
CFST-16	0	900	17.00	5	19.5%	C45	338	458	2538
CFST-17	0	900	17.00	3.6	17.3%	C30	338	458	2406
CFST-18	0	900	17.00	3.6	17.3%	C40	338	458	2504
CFST-19	0	900	17.00	3.6	17.3%	C50	338	458	2540
CFST-20	0	900	17.00	3.6	17.3%	C55	338	458	2544
CFST-21	0	900	17.00	3.6	17.3%	C45	438	458	2552
CFST-22	0	900	17.00	3.6	17.3%	C45	538	458	2650
CFST-23	0	900	17.00	3.6	17.3%	C45	638	458	2707
CFST-24	0	900	17.00	3.6	17.3%	C45	338	558	2572
CFST-25	0	900	17.00	3.6	17.3%	C45	338	658	2764
CFST-26	0	900	17.00	3.6	17.3%	C45	338	758	2989

## 4 Parameter analysis

### 4.1 Eccentricity

**Fig. 7:**
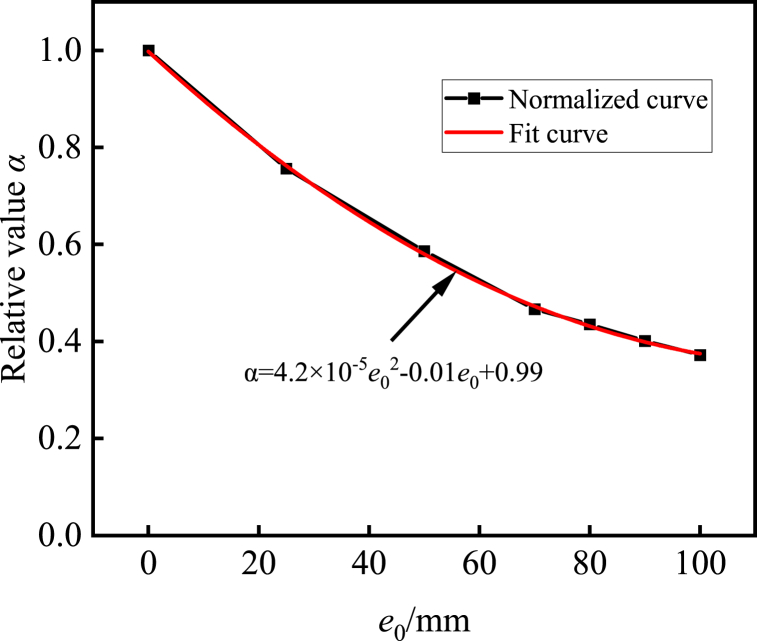
Normalized comparison curve between eccentricity and ultimate bearing capacity.

**Fig. 8:**
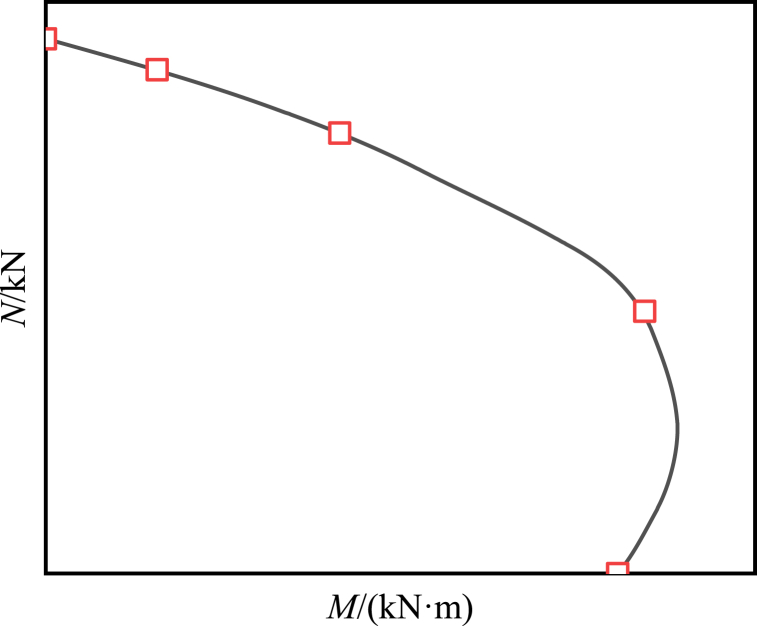
Interaction between axial load and bending moment.

Fig. 7 displays the normalized outcomes with an eccentricity of 0. In Fig. 8, the interplay between axial load and bending moment is depicted. The illustration reveals that as the eccentricity increases, there is a declining trend in the ultimate bearing capacity of the composite cross-shaped steel tube concrete column. After fitting the normalization curve, it is observed that the curve demonstrates a nearly quadratic function relationship. Specifically, for eccentricities smaller than or equal to 70 mm, the bearing capacity degradation is more rapid, whereas for eccentricities larger than 70 mm and up to 100 mm, the bearing capacity degradation is slower. Comparing specimens with an eccentricity of 0 mm to those with eccentricities of 25 mm, 50 mm, 70 mm, 80 mm, 90 mm and 100 mm, the ultimate bearing capacity decreased by 24.4%, 41.4%, 53.3%, 56.5%, 59.9% and 62.8%, respectively. This occurrence can be ascribed to the response observed under eccentric loading, wherein specimens with smaller eccentricity mainly sustain compressive stress. Due to the brittleness of concrete when subjected to compression, it is particularly sensitive to minor eccentricities, resulting in a significant reduction in bearing capacity. Conversely, specimens with larger eccentricity primarily endure tensile stress. Given the low tensile strength of concrete, the contribution of steel to the bearing capacity becomes prominent, leading to a comparatively lesser decline in bearing capacity.

### 4.2 Slenderness ratio

**Fig. 9:**
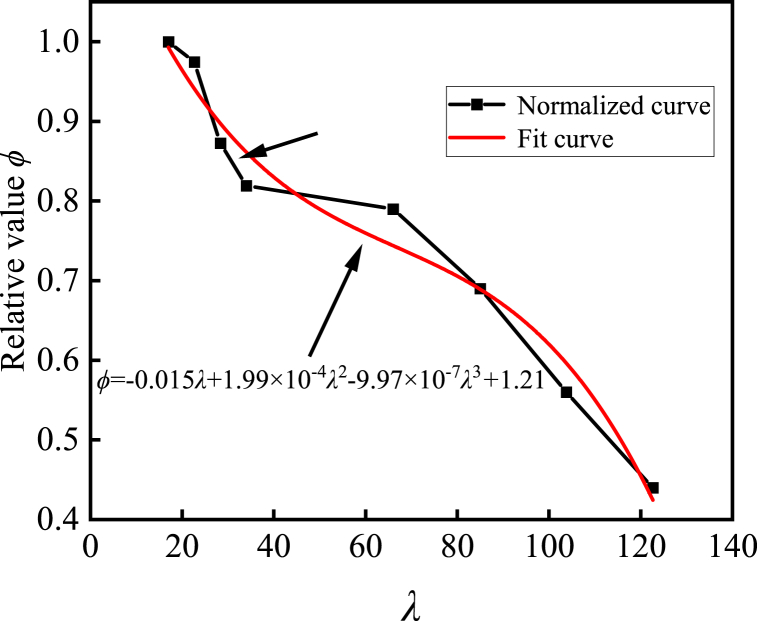
Normalized comparison curve between slenderness ratio and ultimate bearing capacity.

Fig. 9 delineates the normalized outcomes of the peak capacity across specimens exhibiting diverse slenderness ratios, relative to the reference specimen characterized by a slenderness ratio of 17.00. The ratio denoted by *φ* signifies the ultimate bearing capacity of each specimen vis-à-vis the peak bearing capacity of the CFST-5 specimen, serving as a surrogate for the stability coefficient. The graph manifests an initial precipitous decline of approximately 20% in the bearing capacity of the composite cross-shaped steel tube concrete column as the slenderness ratio ascends. Subsequently, a gradual plateau emerges within the slenderness ratio range of 34–66, succeeded by another precipitous descent. Relative to the specimen boasting a slenderness ratio of 17.00, the ultimate bearing capacity undergoes a diminution ranging from 2.6% to 56.0% as the slenderness ratio escalates from 22.66 to 122.64. This decrement is ascribed to the predominance of axial compression in specimens characterized by smaller slenderness ratios, where the plasticity conferred by the steel tube and channel steel counteracts the influence of the slenderness ratio on the bearing capacity. However, when the slenderness ratio exceeds 66, compression bending occurs and instability becomes the primary cause of failure, leading to a rapid decrease in bearing capacity. After fitting the normalized curve, Fig. 9 shows the fitted calculation relationship between the stability coefficient and slenderness ratio, offering theoretical support for the bearing capacity calculation method proposed in the subsequent section.

### 4.3 Section steel content

**Fig. 10:**
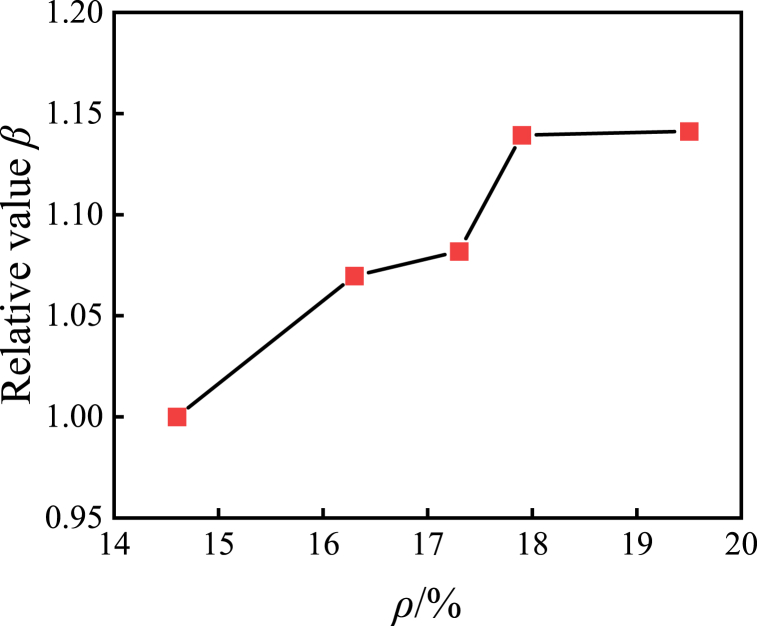
Normalized comparison curve between steel content and ultimate bearing capacity.

Fig. 10 illustrates the normalized outcomes of the ultimate bearing capacity across specimens featuring diverse section steel ratios, relative to specimens characterized by a section steel ratio of 14.6%. The graph elucidates that with the augmentation of section steel content, the ultimate bearing capacity of the composite cross-shaped steel tube concrete column exhibits a gradual augmentation. When comparing specimens with a steel content of 14.6% to those with steel contents of 16.3%, 17.3%, 17.9% and 19.5%, the ultimate bearing capacity increased by 6.9%, 8.1%, 13.9% and 22.6% respectively. This trend can be attributed to the constraint effect of steel tubes, which collaborate with concrete to impose three-dimensional constraints on the concrete core. Consequently, the strain state of concrete improves, leading to an increase in compressive strength. As the steel content of the section increases, the constraint effect becomes more pronounced, resulting in an enhanced compressive capacity of the concrete and ultimately increasing the overall column's ultimate bearing capacity.

### 4.4 Concrete strength grade

**Fig. 11:**
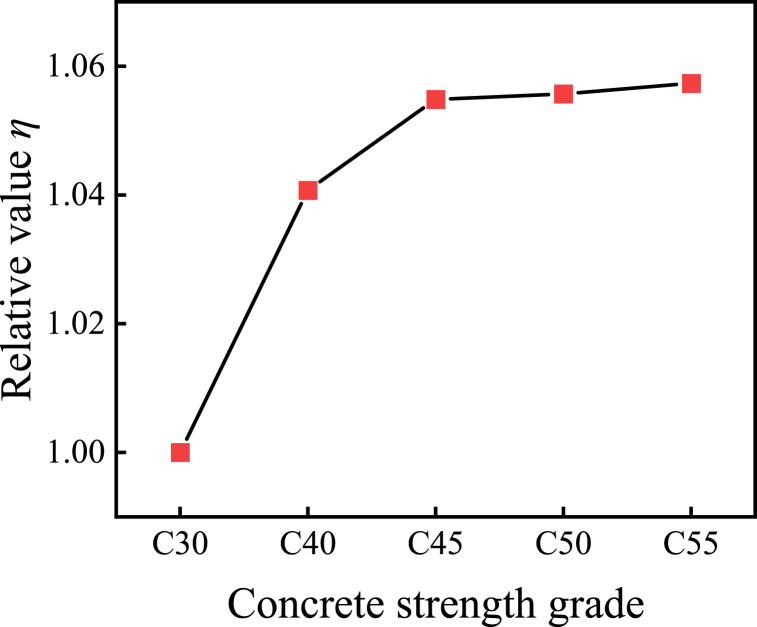
Normalized comparison curve between concrete strength grade and ultimate bearing capacity.

Fig. 11 portrays the normalized ramifications of the ultimate bearing capacity for specimens featuring diverse cross-sectional steel ratios, juxtaposed with C30 specimens. The graph delineates a progressive augmentation in the ultimate bearing capacity of the composite cross-shaped steel tube concrete column with an escalation in concrete strength grade. Notably, for concrete strength grades below C45, there is a discernible surge in the ultimate bearing capacity of the specimen. However, transcending C45, bolstering the concrete strength fails to yield a significant amplification in the ultimate bearing capacity of the specimen. This observation underscores the formidable restraining prowess of steel tubes and channel steel on low-strength concrete, whereas their influence on high-strength concrete is comparatively subdued. Relative to specimens with a concrete strength grade of C30, specimens characterized by concrete strength grades C40, C45, C50 and C55 register a respective augmentation in their ultimate bearing capacity by 4.1%, 5.4%, 5.5% and 5.7%.

### 4.5 Strength of steel

**Fig. 12:**
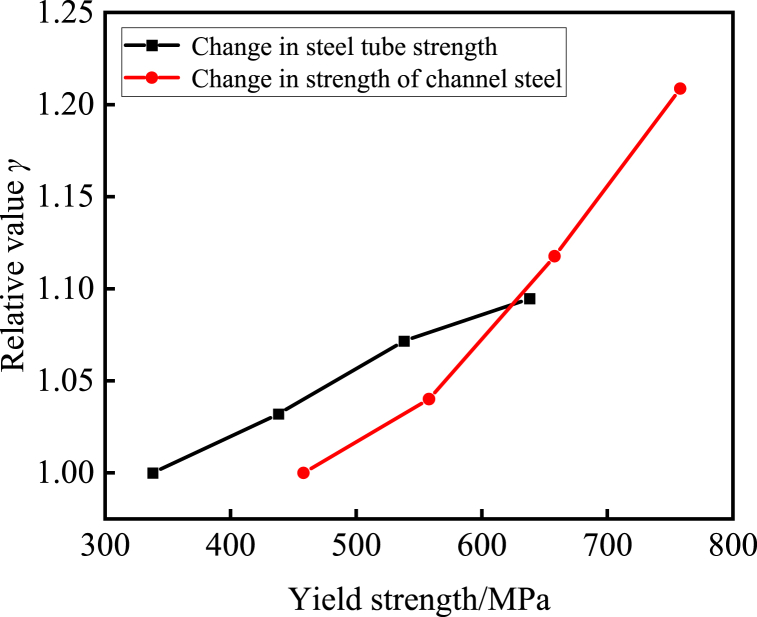
Normalized comparison curve between steel strength and ultimate bearing capacity.

Fig. 12 presents the normalized ramifications of the ultimate bearing capacity for specimens featuring varying steel yield strengths, anchored by specimens endowed with a steel yield strength of 338 MPa. The depiction delineates a progressive surge in the ultimate bearing capacity of the composite cross-shaped steel tube concrete column with the elevation of steel yield strength. Notably, augmenting the strength of the channel steel exerts the most conspicuous impact on the bearing capacity of the specimen. This phenomenon can be elucidated by the strategic placement of the channel steel along the periphery of the specimen, which not only imposes constraints on the concrete encapsulated within but also, to a certain degree, curtails the movement of concrete within the steel tube. Comparative scrutiny reveals that in contrast to the reference specimens featuring a yield strength of 338 MPa for steel tubes and 458 MPa for channel steel, the ultimate bearing capacity of the specimens amplifies by 3.1%, 7.1%, 9.4%, and 4.0%, 11.7% and 20.8% for every 100 MPa increment in yield strength for steel tubes and channel steel, respectively.

## 5 Calculating method for column's bearing capacity

### 5.1 Determining the ultimate capacity under axial compression

In antecedent inquiries, Wu et al. [29,30] delved into an intricate unified theory-driven approach for evaluating the load-bearing capacity of distinctive-shaped concrete columns fabricated utilizing steel tubes. They also scrutinized the impacts of various void configurations on the amalgamated calculations. Consequently, this study simplifies the axial compression behavior of combined cross-shaped concrete columns containing steel tubes into five distinct configurations of rectangular steel tube concrete combinations. By applying Han Linhai's theory on confined concrete within rectangular steel tubes [31], a computational approach for determining the axial compression load-bearing capacity of combined cross-shaped concrete columns made of steel tubes is presented, as depicted in Eq. (4):

(4) $$P_{0}=\varphi \left(A_{\text{cs}}\cdot f_{\text{ycs}}+A_{\text{ss}}\cdot f_{\text{yss}}+A_{\text{ccs}}\cdot f_{\text{ccs}}+A_{\text{css}}\cdot f_{\text{css}}\right)$$

where *P*_0_ is the maximum capacity of the cross shaped CFST column. *φ* is the axial compressive stability coefficient, calculated according to Fig. 9. *A*_cs_ and *A*_ss_ are the total area of channel steel and square steel tubes, respectively. *F*_ycs_ and *f*_yss_ are the yield strength of channel steel and square steel tubes, respectively. *A*_ccs_ and *A*_css_ respectively represent the total area of concrete in the core area of channel steel and square steel tubes. *F*_ccs_ and *f*_css_ are the compressive strength of the confined concrete in the core area of channel steel and square steel tubes, respectively, calculated according to Eq. (5) and Eq. (6).

(5) $$f_{\text{ccs}}=\left[1+\left(-0.0135\zeta_{c}^{2}+0.1\zeta_{c}\right){\left(\frac{24}{f_{c}}\right)}^{0.45}\right]f_{c}$$

(6) $$f_{\text{css}}=\left[1+\left(-0.0135\zeta_{s}^{2}+0.1\zeta_{s}\right){\left(\frac{24}{f_{c}}\right)}^{0.45}\right]f_{c}$$

where *ξ* is the hoop coefficient, which is calculated according to Eq. (7) for the channel steel part and Eq. (8) for the square steel tube part.

(7) $$\zeta_{c}=\frac{A_{\text{cs}}}{A_{\text{ccs}}}\cdot \frac{f_{\text{ycs}}}{f_{\text{ccs}}}$$

(8) $$\zeta_{s}=\frac{A_{\text{ss}}}{A_{\text{css}}}\cdot \frac{f_{\text{yss}}}{f_{\text{css}}}$$

### 5.2 Determination of ultimate load-bearing capacity under eccentric compression

**Fig. 13:**
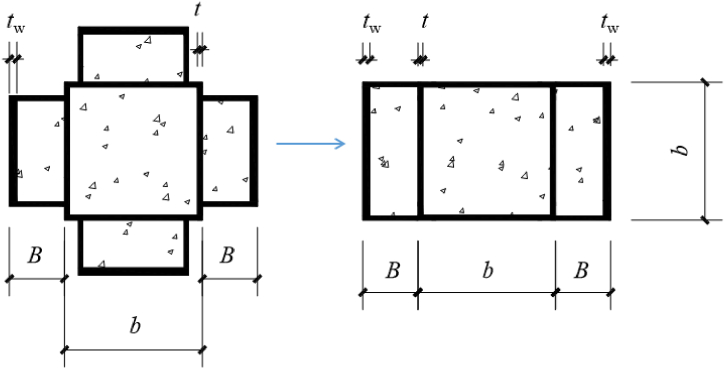
Model simplification.

Evaluating the bending capacity of the uniquely shaped steel tube concrete column is intricate owing to the irregularity of the section's shape. This complexity arises from challenges in computing the geometric parameters and bending load-bearing capacity of the cross-section. Furthermore, the interplay between the steel tube and the central concrete introduces additional intricacies into the calculation process [[32], [33], [34]]. To simplify the calculation and ensure safety in engineering design, a streamlined approach is proposed. The composite cross shaped steel tube concrete column is simplified to resemble a rectangular steel tube concrete eccentric compression column without any channel steel (as depicted in Fig. 13). This method overlooks the influence of channel steel concrete on both sides and concentrates on the effect of channel steel concrete in the compression/tension region. Several assumptions are made within this framework.

(1) The steel tube wall did not experience local buckling.
(2) The steel tube undergoes full section yielding (i.e., yielding in tension in the tensile zone and yielding in compression in the compressive zone).
(3) The compressive strength of the concrete in the compression zone adheres to the standard value *f*_c_ of the axial compressive strength of the cylinder.
(4) Neglecting the tensile effect of concrete.

The stress examination employs the limit equilibrium theory and is derived following the calculation approach for determining the ultimate load-bearing capacity of eccentrically compressed rectangular section members outlined in the “Design Code for Concrete Structures" in China, as shown in Eqs. (9), (10)):

(9) $$N_{u}=f_{c}bx+\left[\left(b-2t\right)t+\left(b-2t_{w}\right)h_{w}+2tx\right]f_{y}$$

(10) $$N_{u}e=f_{c}bx\left(b+2B-\frac{x}{2}\right)+\left(b-2t\right)\left(x-B\right)tf_{y}+\left(b-2t_{w}\right)\left(b+2B\right)h_{w}$$

Here *N*_u_ denotes the ultimate capacity under eccentric compression, *e* represents the eccentricity, *b* signifies the length of the steel tube side, *t* indicates the thickness of the steel tube, *B* denotes the length of the short side of the channel steel, *h*_w_ stands for the thickness of the long side of the channel steel, *f*_y_ represents the yield strength of the steel and *x* signifies the relative height of the compression zone.

### 5.3 Verification of calculation results

Table 4 depicts the comparison of calculation outcomes obtained from the test specimen and finite element specimen, employing the previously mentioned calculation method. The table clearly indicates that the average value of the ultimate bearing capacity ratio, computed using the eccentric bearing capacity formula, closely corresponds to the test/finite element results with an average of *μ* = 1.135. The variance is *D* = 0.025 and the coefficient of variation is *CV* = 0.022. Similarly, the average value of the ultimate bearing capacity ratio, determined from the axial bearing capacity calculation formula, aligns well with the experimental/finite element results at an average of *μ* = 1.018. The variance is *D* = 0.004 and the coefficient of variation is *CV* = 0.004. These values demonstrate a close concurrence with the experimental results, albeit with a slight underestimation, ensuring a certain level of safety. The findings conclusively affirm the high applicability of the proposed method for calculating the ultimate bearing capacity under pressure, as elucidated in this paper. It is noteworthy that the bearing capacity calculation method proposed in this paper is applicable to the dimensions of the specimens in this study. Further validation is necessary for computing the bearing capacity of specimens of varying sizes.

**Table 4:** Comparing calculated and experimental bearing capacities.

Column no.	Tested peak load *P*_t_ (kN)	Calculated peak load *P*_0_ (kN)	*P*_t_/*P*_0_
spa-25	1871	1644	1.14
spa-50	1450	1211	1.20
spb-25	1842	1623	1.13
spb-50	1254	1108	1.13
spc-25	1607	1432	1.12
spc-50	1190	1022	1.16
CFST-1	1155	1012	1.14
CFST-2	1077	980	1.10
CFST-3	992	857	1.16
CFST-4	921	810	1.14
CFST-5	2473	2371	1.04
CFST-6	2410	2254	1.07
CFST-7	2158	2106	1.02
CFST-8	2026	1958	1.03
CFST-9	1953	1933	0.99
CFST-10	1706	1688	0.99
CFST-11	1384	1411	1.02
CFST-12	1088	1109	1.02
CFST-13	2224	2176	1.02
CFST-14	2379	2299	1.03
CFST-15	2534	2418	1.05
CFST-16	2538	2533	1.00
CFST-17	2406	2141	1.12
CFST-18	2504	2297	1.09
CFST-19	2540	2444	1.04
CFST-20	2544	2516	1.01
CFST-21	2552	2583	0.99
CFST-22	2650	2695	0.98
CFST-23	2707	2788	0.97
CFST-24	2572	2689	0.96
CFST-25	2764	2799	0.99
CFST-26	2989	3105	0.96

## 6 Conclusions

(1) Employing the ABAQUS finite element analysis software, a meticulous simulation and analysis of composite cross-shaped steel tube concrete columns subjected to eccentric compression was conducted. The resultant calculated outcomes intricately mirror the experimental findings.
(2) The ultimate bearing capacity of the composite cross-shaped steel tube concrete column exhibits a decrement with escalating eccentricity and slenderness ratio, while augmenting with elevated steel content and concrete strength. Remarkably, the specimen's ultimate bearing capacity experiences a rapid deterioration for eccentricities less than 70 mm, and a swift augmentation for concrete strength grades below C45.
(3) Bolstering the strength of channel steel emerges as a more potent strategy for enhancing the load-bearing capacity of specimens compared to elevating the strength of steel tubes.
(4)Grounded on the principles of steel tube confined concrete, the proposed methodology for computing the ultimate bearing capacity of composite steel tube concrete columns under axial and eccentric compression manifests commendable applicability and safety assurance.

## CRediT authorship contribution statement

**Mingjian Yang:** Formal analysis, Data curation. **Desun Yu:** Formal analysis, Data curation. **Qirong Qiu:** Formal analysis, Data curation. **Yong Yu:** Writing – review & editing, Funding acquisition, Conceptualization.

## Declaration of competing interest

The authors declare that they have no known competing financial interests or personal relationships that could have appeared to influence the work reported in this paper.
